# Changes in HIV Testing Utilization Among Chinese Men Who Have Sex With Men During the COVID-19 Pandemic in Shenzhen, China: An Observational Prospective Cohort Study

**DOI:** 10.3389/fmed.2022.842121

**Published:** 2022-06-09

**Authors:** Kechun Zhang, Siyu Chen, Paul Shing-fong Chan, Yuan Fang, He Cao, Hongbiao Chen, Tian Hu, Yaqi Chen, Xiaofeng Zhou, Zixin Wang

**Affiliations:** ^1^Longhua District Center for Disease Control and Prevention, Shenzhen, China; ^2^JC School of Public Health and Primary Care, The Chinese University of Hong Kong, Hong Kong, Hong Kong SAR, China; ^3^Department of Health and Physical Education, The Education University of Hong Kong, Hong Kong, Hong Kong SAR, China

**Keywords:** HIV testing utilization, men who have sex with men, COVID-19, observational prospective cohort study, predictors, China

## Abstract

**Background:**

The Coronavirus Diseases 2019 (COVID-19) directly affects HIV prevention and sexual health services utilization among men who have sex with men (MSM). This study investigated changes in human immunodeficiency virus (HIV) testing utilization among MSM before and after the COVID-19 pandemic received initial control in Shenzhen, China.

**Methods:**

This study was a sub-analysis of a prospective observational cohort study conducted among MSM in Shenzhen, China between August 2020 and May 2021. Participants were recruited through outreaching in gay venues, online recruitment, and peer referral. Participants completed a baseline online survey between August and September 2020 and a follow-up online survey between April and May 2021. This study was based on 412 MSM who reported to be HIV-negative/unknown sero-status at baseline, 297 (72.1%) of them completed the follow-up online survey. Multilevel logistic regression models (level 1: sources of recruitment; level 2: individual participants) were fitted.

**Results:**

When comparing follow-up data with baseline data, a significant increase was observed in the uptake of any type of HIV testing (77.9% at Month 6 vs. 59.2% at baseline, *p* < 0.001). After adjusting for age group, education level, current employment status and monthly personal income, two predisposing factors were associated with higher uptake of HIV testing during the follow-up period. They were: (1) condomless anal intercourse with male non-regular male sex partners at follow-up only (AOR: 5.29, 95%CI: 1.27, 22.01) and (2) sanitizing before and after sex at baseline (AOR: 1.26, 95%CI: 1.02, 1.47). Regarding enabling factors, utilization of HIV testing (AOR: 3.90, 95%CI: 2.27, 6.69) and STI testing (AOR: 2.43, 95%CI: 1.20, 4.93) 6 months prior to the baseline survey was associated with higher uptake of HIV testing during the follow-up period. Having the experience that HIV testing service providers reduced service hours during the follow-up period was also positively associated with the dependent variable (AOR: 3.45, 95%CI: 1.26, 9.41).

**Conclusions:**

HIV testing utilization among MSM might rebound to the level before the COVID-19 outbreak after the pandemic received initial control in China. This study offered a comprehensive overview to identify potential reasons that can influence the uptake of HIV testing among Chinese MSM.

## Introduction

The human immunodeficiency virus (HIV) epidemic among men who have sex with men (MSM) remains out of control across countries (1). A systematic review showed an overall HIV prevalence of 5.7% among MSM in China (1), whereas the HIV incidence in this group was 5.6 per 100 person-year (2). High coverage of HIV testing (i.e., >90%) among the at-risk population is the first and crucial step to achieve the 90-90-90 targets established by the Joint United Nations Programme on HIV/AIDS, which sheds the hope of ending the global HIV epidemic in 2030 (3). International health authorities recommend MSM take up HIV testing every 6 months (4, 5). However, HIV testing coverage remained low among MSM in China, as only 60% of Chinese MSM received any type of HIV testing in the past year (6).

The Coronavirus Diseases 2019 (COVID-19) is a serious health threat worldwide (7). The COVID-19 pandemic and its control measures (i.e., lockdown, physical distancing, and closure of business) had direct impacts on HIV prevention and sexual health services for MSM. In Japan, the number of HIV tests performed by public health centers significantly declined in the second quarter of 2020 (9,584 vs. 35,908 in the year-before period) (8). A similar situation was observed in Melbourne, Australia, where the number of HIV testing decreased from 16,367 in 2019 to 11,270 in 2020 with a 31% reduction (9). An online survey of a global sample of MSM showed that only 30 and 19% of participants had similar levels of access to facility-based HIV testing and HIV self-testing during the pandemic compared to their situation in 2019 (10). In the United States, 18.8% of MSM decreased access to HIV testing and 5.6% had trouble of getting HIV testing after the COVID-19 outbreak (11). Two cross-sectional studies investigated the impact of COVID-19 on HIV testing service utilization among Chinese MSM (12, 13). During the pandemic, 56.8% of Chinese MSM had trouble of accessing HIV testing services (12). As compared to the time before COVID-19, the utilization of facility-based HIV testing declined but the use of HIV self-testing increased (13). In China, the government implemented a lockdown in Wuhan city from January 23 to April 8, 2020. Although there was no lockdown, most Chinese cities including Shenzhen started to implement strict control measures such as the closure of non-emergency health services and non-essential business activities by the end of January 2020. Starting from May 2020, Shenzhen and most parts of China gradually relieved the control measures and resumed non-emergency health services. These services resumed to normal around July 2020 in Shenzhen. Therefore, as parts of non-emergency health services, HIV testing and other sexual health services for MSM were mostly affected by the COVID-19 control measures between January and May 2020. At the time of our baseline survey (August to September 2020), the COVID-19 pandemic in China received initial control. MSM may increase sexual activities after COVID-19 control measures were relaxed. It is hence important to know whether the HIV testing service utilization would rebound once the COVID-19 epidemic was under control.

It is also helpful to understand factors associated with HIV testing utilization in the “post-pandemic era.” The Predisposing, Reinforcing and Enabling Constructs in Educational/Ecological Diagnosis and Evaluation (PRECEDE) model was used as the theoretical framework (14). This model posits that health behaviors (i.e., utilization of HIV testing) are influenced by predisposing factors (characteristics that motivate behavior, such as perceptions and other psychosocial variables), enabling factors (characteristics that facilitate or hinder the performance of a behavior), and reinforcing factors (rewards and punishments). Regarding predisposing factors, the COVID-19 pandemic increased some perceived barriers to use HIV testing, such as the fear of going to hospitals because of COVID-19, concerns about COVID-19 infection or having close contact with COVID-19 patients during HIV testing, and perceptions that health workers were reluctant to serve them during the pandemic (8, 15–18). Moreover, the presence of sexual risk behaviors was a strong predictor of HIV testing utilization among MSM (19, 20). An increase in sexual risk behaviors after the pandemic was under control might motivate them to take up HIV testing. Regarding enabling factors, we considered access to other health services and barriers to use HIV testing caused by COVID-19 and its control measures (i.e., suspension of testing services, history of COVID-19 infection, and centralized/home quarantine). Closure of facilities providing HIV testing services, shortage of medical staff providing HIV testing, suspension of public transportation, and lockdown/travel restrictions were barriers to access HIV testing services during the pandemic (8, 15–18). Regarding reinforcing factors, Chinese MSM tend to have large and dense social networks, and most of them are heavily involved in the community and have strong ties to each other (21). Social norms are social attitudes of approval or disapproval that are specific to what should be or should not be done in the context of health. Perceived social norm supporting HIV testing was a facilitator for MSM to take up HIV testing (22). MSM may value the opinions of their male sex partners or peers. Therefore, being suggested not to take up HIV testing during the pandemic by male partners or peers would hinder MSM to receive such service.

To address the knowledge gaps, we conducted a longitudinal observational study to investigate the changes in HIV testing service utilization among MSM during the COVID-19 pandemic. We also investigated factors associated with HIV testing utilization during the follow-up period, including socio-demographics, predisposing factors (change in sexual behaviors, perceptions, and psychosocial variables), enabling factors (access to other health services and reinforcing factors (the suggestions made by male sex partners or peers).

## Methods

### Study Design

This study was a sub-analysis of a prospective observational cohort study conducted among MSM in Shenzhen, China between August 2020 and May 2021. Participants completed a baseline survey between August and September 2020 and a follow-up survey between April and May 2021.

### Participants and Data Collection

Participants of the cohort study were: (1) Chinese-speaking men living in Shenzhen, (2) aged 18 years or above, (3) had oral or anal intercourse with at least one man in the past year, and (4) willing to leave contact information and complete the follow-up survey. We recruited participants through multiple sources. Trained and experienced fieldworkers approached prospective participants in venues frequently visited by MSM (i.e., bars, parks, and bathhouses) at different time slots during weekdays and weekends. An online outreaching was also conducted by periodically posting study information on two commonly used social media platforms in China (Weibo and the official WeChat account). Recruitment was supplemented by peer referrals. On-site or through telephone/live chat applications, fieldworkers briefed prospective participants about the study details and invited them to add the project's official WeChat account. Fieldworkers screened the eligibility of prospective participants and assured participants that their identifiable information (e.g., their WeChat account) would be kept confidential, they had the right to discontinue participation in the study at any time, and their refusal or withdrawal from the study would not have any consequences. The project's official WeChat account would delink with participants' WeChat accounts after the project ended. Participants signed an electronic consent form sent by WeChat. Participants were also informed that their electronic consent form would be separated from the database and stored in a password-protected computer. The same approach to protect participants' personal information was used in a published study (23).

**Figure 1:**
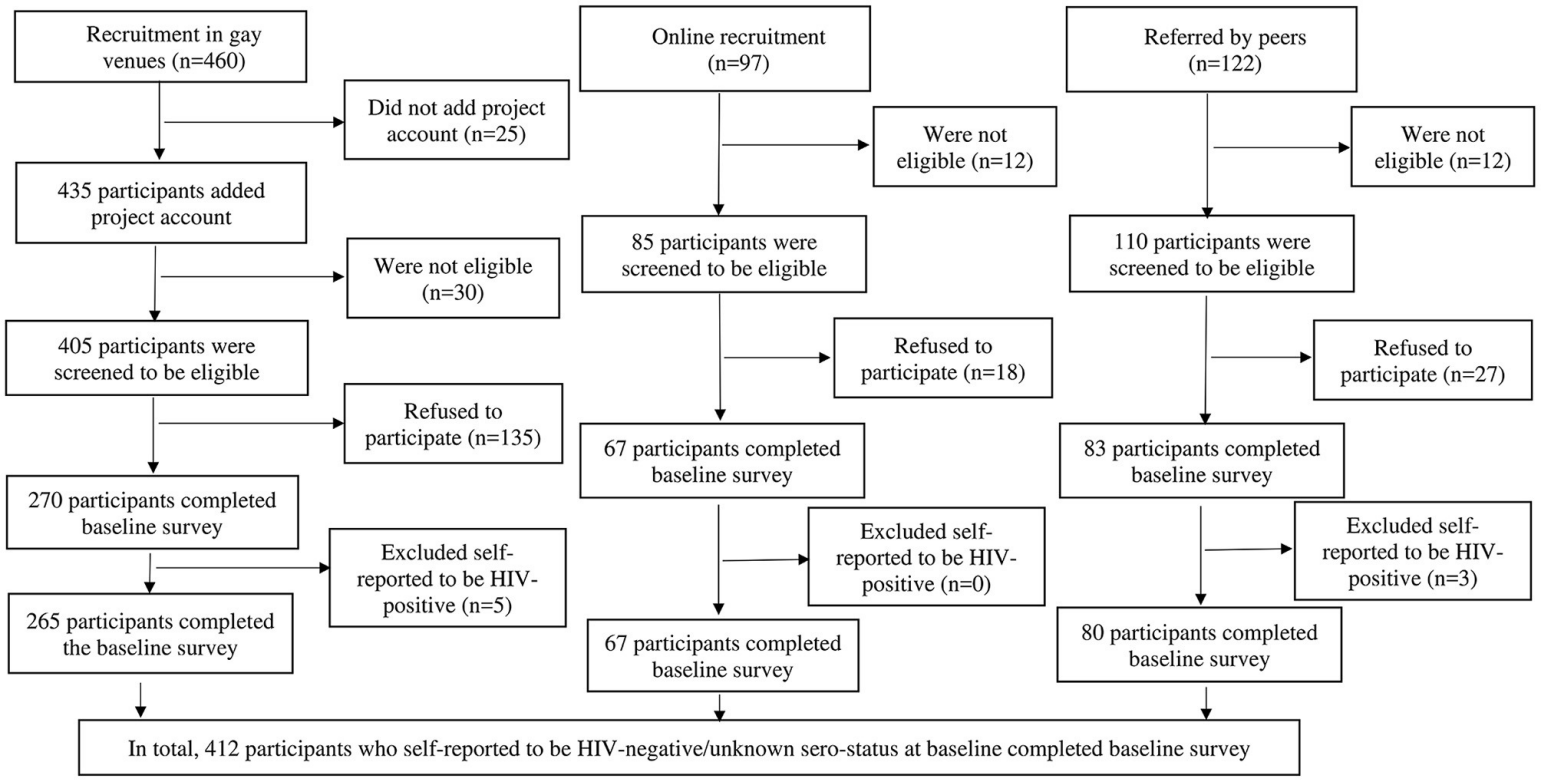
Flowchart of data collection.

We developed an online self-administered questionnaire using Questionnaire Star, a commonly used online survey platform in China. Quick response codes were generated and sent to consented participants through WeChat. Participants scanned the quick response codes to complete the survey. Each mobile device was only allowed to access the online questionnaire once to avoid duplicate responses. The baseline survey had 105 items (about 20 items per page for five pages), which took about 20 min to complete. The follow-up survey was shorter (80 items), which took about 15 min to complete. We assigned a unique study ID to each participant to link their baseline and follow-up data. The Questionnaire Star tool performed completeness checks before the questionnaire was submitted. Participants were able to review and change their responses through a “Back” button. An electronic coupon of 20 Chinese Yuan (the US $3.0) was sent to participants upon completion of each survey. All data was stored in the online server of Questionnaire Star and protected by a password. Only the principal investigator of the study had access to the database. The fieldworkers approached 460 prospective participants in gay venues, 435 added the project's official WeChat account, 405 were screened eligible through WeChat, 135 refused to participate and 270 completed the baseline survey. Regarding online recruitment, 97 prospective participants contacted the fieldworkers, 85 were screened to be eligible, 18 refused to participate and 67 completed the baseline survey. Among 122 prospective participants referred by peers, 110 were screened to be eligible, 27 refused to participate and 83 completed the baseline survey. In sum, 420 participants completed the baseline survey. We excluded eight participants who self-reported to be HIV-positive at the baseline as this study focused on the changes in HIV testing behaviors. This study was based on 412 participants who self-reported to be HIV-negative/unknown sero-status at baseline (venue: *n* = 265, online: *n* = 67, and referral: *n* = 80). A flowchart of recruitment was shown in Figure 1. At Month 6, 297 (72.1%) participants completed the follow-up survey. Ethics approval was obtained from the Longhua District Center for Disease Control and Prevention (CDC) (reference: 2021009).

### Measures

#### Design of the Questionnaire

The questionnaire was developed by a panel that included a public health researcher, an epidemiologist, a psychologist, a member of the MSM community, and a member of a community-based organization. The questionnaire was tested among 10 local MSM. These 10 MSM did not participate in the cohort. Based on their feedback the questionnaire was finalized by the panel.

#### Sociodemographic Characteristics

At baseline, participants were asked to report on sociodemographic characteristics, including age, current relationship status, highest education level attained, current employment status, monthly personal income, and sexual orientation.

#### Utilization of HIV Testing Services

Utilization of the following types of HIV testing in the past 6 months was recorded at both baseline and follow-up surveys, including: (1) HIV testing at community-based organizations in Shenzhen, (2) HIV testing at public hospitals or CDC in Shenzhen, (3) HIV testing at private hospitals in Shenzhen, (4) HIV testing at other organizations in Shenzhen, (5) HIV testing outside Shenzhen, and (6) home-based HIV self-testing. These measurements were adapted from previous studies among Chinese MSM (21, 24, 25).

#### Predisposing Factors

Participants reported the presence of condomless anal intercourse with regular male sex partners, non-regular male sex partners, and male sex workers, and sexualized drug use in the last 6 months prior to the baseline and follow-up surveys. Regular male sex partners were defined as their lovers or stable boyfriends, while non-regular male sex partners were defined as casual sex partners. Sexualized drug use was defined as the use of any of the following psychoactive substances [i.e., ketamine, methamphetamine, cocaine, cannabis, ecstasy, Dormicum/Halcion/Erimin 5/non-prescription hypnotic drugs, heroin, codeine from cough suppressants, amyl nitrates (poppers), γ-hydroxybutyrate (G water), and Foxy] before or during sexual intercourse (19, 26). The measurements of these sexual risk behaviors were also adapted from previous studies targeting Chinese MSM (27–29). Four variables were created by comparing the presence of such behaviors at follow-up vs. baseline (response categories: 1 = no such behavior either at baseline or follow-up, 2 = with such behavior at baseline only, 3 = with such behavior both at baseline and follow-up, and 4 = with such behavior at follow-up only).

Participants also reported the frequency of using COVID-19 preventive measures during sexual behaviors 6 months prior to the baseline survey (response categories: 1 = never, 2 = seldom, 3 = sometimes, 4 = often, 5 = always). These preventive measures were: (1) only having sex with your partner who has had sex with you before, (2) avoiding group sex, (3) only having sex at home, (4) asking your partner if they have COVID-19 symptoms, (5) avoiding kissing during sex, (6) washing hands before and after sex, and (7) sanitizing before and after sex.

At baseline, participants were also asked whether they were concerned about being infected with COVID-19 when taking up HIV testing (response categories: from 1 = strongly disagree to 5 = strongly agree). Two single items measured the perceived effectiveness of COVID-19 control measures taken by organizations providing HIV testing was measured (response categories: from 1 = not effective at all to 5 = very effective) and the level of panic of COVID-19 (response categories: from 1 = not at all to 5 = very high).

#### Enabling Factors

Participants reported the utilization of sexually transmitted infections (STI) testing, pre-exposure prophylaxis, and other HIV/STI prevention services 6 months prior to the baseline and follow-up surveys. Experiences about HIV testing service providers suspending their services or reducing service hours, difficulty in obtaining HIV self-testing kits, history of home/centralized quarantine, and avoiding unnecessary travel or going to crowded places were recorded at follow-up.

#### Reinforcing Factors

At follow-up, participants were asked whether their male sex partners or peers suggested them not take up HIV testing in the past 6 months.

### Statistical Analysis

Baseline characteristics of participants who completed the follow-up survey and those who were loss-to-follow-up were compared using chi-square tests (for categorical variables) or independent sample *t*-tests (for continuous variables). We filled the missing outcome values at Month 6 with multiple imputations. Markov Chain Monte Carlo methods were used for data with the arbitrary pattern of missing values, while Monotone methods were used for data having a monotone pattern of missing values. Predictors included baseline background characteristics and baseline values of the variables with missing values at Month 6. Utilization of any type and specific type of HIV testing and sexual behaviors at baseline and follow-up were compared using McNemar tests. Using uptake of any type of HIV testing during the follow-up period as the dependent variable, and sociodemographic characteristics as independent variables, crude odds ratios (OR) were obtained using multilevel logistic regression models (level 1: sources for recruitment; level 2: individual participants). After adjusting for background characteristics in univariate analysis, the associations between independent variables of interest (predisposing, enabling, and reinforcing factors) and the dependent variable were investigated by using multivariate multilevel logistic regression models (level 1: sources for recruitment; level 2: individual participants). Random intercept models were used to allow the intercept of the regression model to vary across sources for recruitment, which could account for intra-correlated nested data. Each multivariate multilevel logistic regression model involved one of the independent variables of interest and age group, education level, current employment status, and monthly personal income. In the literature, age, education level, occupation, and monthly income were associated with HIV testing uptake among Chinese MSM (6, 24, 30, 31). Adjusted odds ratios (AOR) were obtained. Multilevel logistic/linear regression models are commonly used in studies with cluster sampling methods (32–34). SPSS 26.0 (Chicago, IL, USA) was used for data analysis, and *p* < 0.05 was considered statistically significant.

## Results

### Baseline and Follow-Up Characteristics of Participants

The majority of the participants were aged 18–30 years (76.5%), currently single (80.4%), received tertiary education (60.9%), full-time employed (70.6%), and self-identified as homosexual (71.6%). Significant differences were found in the following baseline characteristics between those who completed the follow-up survey (*n* = 297) and those who were loss-to-follow-up (*n* = 115). These baseline characteristics were: (1) sexual orientation (*p* < 0.001), (2) performed HIV testing outside Shenzhen (*p* = 0.03), (3) uptake of any type of HIV testing (*p* = 0.02), (4) COVID-19 preventive measures during sexual behaviors (*p* = 0.001 to 0.009), and (5) perceived effectiveness of COVID-19 control measures taken by organizations providing HIV testing (*p* = 0.02) (Table 1).

**Table 1:** Baseline characteristics of participants.

	**All participants** **(*n* = 412)**	**Follow-up at Month 9** **(*n* = 297)**	**Loss to follow-up** **(*n* = 115)**	***P*** **-values**
	***n*** **(%)**	***n*** **(%)**	***n*** **(%)**	
**Sociodemographic**				
**Age group (years)**				
18–24	130 (31.6)	87 (29.3)	43 (37.4)	0.18
25–30	185 (44.9)	139 (46.8)	46 (40.0)	
31–40	74 (18.0)	57 (19.2)	17 (14.8)	
>40	23 (5.5)	14 (4.7)	9 (7.8)	
**Current relationship status**				
Currently single	331 (80.4)	234 (78.8)	97 (84.3)	0.41
Married or cohabited with a man	66 (16.0)	52 (17.5)	14 (12.2)	
Married or cohabited with a woman	15 (3.6)	11 (3.7)	4 (3.5)	
**Highest education level attained**				
Junior high or below	52 (12.6)	35 (11.8)	17 (14.8)	0.15
Senior high or equivalent	93 (22.6)	60 (20.2)	33 (28.7)	
College or above	251 (60.9)	191 (64.3)	60 (52.2)	
Others	16 (3.9)	11 (3.7)	5 (4.3)	
**Current employment status**				
Full-time	291 (70.6)	216 (72.7)	75 (65.2)	0.13
Part-time/unemployed/retired/students/others	121 (29.4)	81 (27.3)	40 (34.8)	
**Monthly personal income, Chinese Yuan (US dollar)**				
No fixed income	38 (9.2)	27 (9.1)	11 (9.6)	0.12
Below 3,000 (461.4)	33 (8.0)	22 (7.4)	11 (9.6)	
3,000–4,999 (461.4–768.9)	91 (22.2)	61 (20.5)	30 (26.1)	
5,000–6,999 (769.1–1076.5)	86 (20.9)	56 (18.9)	30 (26.1)	
7,000–9,999 (1076.7–1537.9)	69 (16.7)	57 (19.2)	12 (10.4)	
10,000 or above (1538.1)	76 (18.4)	61 (20.5)	15 (13.0)	
Refuse to disclose	19 (4.6)	13 (4.4)	6 (5.2)	
**Sexual orientation**				
Homosexual	295 (71.6)	233 (78.5)	62 (53.9)	**<0.001**
Bisexual	83 (20.1)	51 (17.2)	32 (27.8)	
Heterosexual	14 (3.4)	3 (1.0)	11 (9.6)	
Uncertain	20 (4.9)	10 (3.3)	10 (8.7)	
**Recruitment sources**				
Outreaching in gay venues	265 (64.3)	190 (64.0)	75 (65.2)	0.93
Online recruitment	67 (16.3)	48 (16.2)	19 (16.5)	
Peer referral	80 (19.4)	59 (19.9)	21 (18.3)	
**Predisposing factors**				
**Sexual behaviors in the past 6 months (February to July 2020)**, ***n*** **(%), Yes**				
Condomless anal intercourse with regular male sex partners	99 (24.0)	73 (24.6)	26 (12.6)	0.68
Condomless anal intercourse with non-regular male sex partners	51 (12.4)	38 (12.8)	13 (11.3)	0.68
Condomless anal intercourse with male sex workers	10 (2.4)	5 (1.7)	5 (4.3)	0.15
Sexualized drug use	50 (12.1)	38 (12.8)	12 (10.4)	0.51
**COVID-19 preventive measures during sexual behaviors (February to July 2020)**, ***n*** **(%) always/often**				
Only having sex with my partner who has had sex with me before	138 (33.5)	111 (37.4)	27 (23.5)	**0.007**
Avoiding group sex	170 (41.3)	136 (45.8)	34 (29.6)	**0.003**
Only having sex at home	170 (41.3)	135 (45.5)	35 (30.4)	**0.005**
Asking your partner if they have COVID-19 symptoms	109 (26.5)	89 (30.0)	20 (17.4)	**0.009**
Avoiding kissing during sex	103 (25.0)	85 (28.6)	18 (15.7)	**0.006**
Washing hands before and after sex	247 (60.0)	193 (65.0)	54 (47.0)	**0.001**
Sanitizing before and after sex	170 (41.3)	136 (45.8)	34 (29.6)	**0.003**
**Psychosocial variables related to COVID-19 and HIV testing**				
Worry about being infected with COVID-19 when undertaking HIV testing, n (%) strongly agree/agree	162 (39.3)	118 (39.7)	44 (38.3)	0.78
Mean (SD)	3.2 (1.3)	3.3 (1.3)	3.0 (1.4)	0.06
Perceived effectiveness of COVID-19 control measures taken by organizations providing HIV testing, *n* (%) very effective/relatively effective	235 (57.0)	177 (59.6)	58 (50.4)	0.09
Mean (SD)	3.5 (1.1)	3.6 (1.1)	3.3 (1.2)	**0.02**
Level of panic of COVID-19 infections, *n* (%) very high/relatively high	135 (32.8)	100 (33.7)	35 (30.4)	0.53
Mean (SD)	3.1 (1.1)	3.1 (1.0)	3.0 (1.2)	0.37
**Enabling factors**				
**Use of different types of HIV testing in the past 6 months (February to July 2020)**, ***n*** **(%) Yes**				
HIV testing at community-based organization in Shenzhen	47 (11.4)	38 (12.8)	9 (7.8)	0.16
HIV testing at public hospitals or CDC in Shenzhen	85 (20.6)	62 (20.9)	23 (20.0)	0.84
HIV testing at private hospitals in Shenzhen	20 (4.9)	17 (5.7)	3 (2.6)	0.19
HIV testing at other organizations in Shenzhen	38 (9.2)	28 (9.4)	10 (8.7)	0.82
HIV testing outside Shenzhen	74 (18.0)	61 (20.5)	13 (11.3)	**0.03**
Home-based HIV self-testing	176 (42.7)	134 (45.1)	42 (36.5)	0.11
Any type of HIV testing	244 (59.2)	186 (62.6)	58 (50.4)	**0.02**
**Utilization of other HIV/STI prevention services in the past 6 months (February to July 2020)**, ***n*** **(%), Yes**				
Testing for STI	101 (24.5)	78 (26.3)	23 (20.0)	0.19
Other HIV/STI prevention services ^a^	151 (36.7)	114 (38.4)	37 (32.2)	0.24
Pre-exposure prophylaxis	28 (6.8)	20 (6.7)	8 (7.0)	0.94

a *Other HIV/STI prevention services: condom distribution, peer education, HIV/STI promotion leaflets and lectures, and HIV/STI prevention knowledge via the Internet or social media. The bold values indicate the values of p < 0.05 which are statistically significant*.

### Utilization of HIV Testing at Baseline and Follow-Up Surveys

When comparing follow-up data with baseline data, a significant increase was observed in the uptake of any type of HIV testing (77.9% at Month 6 vs. 59.2% at baseline, *p* < 0.001), HIV testing at community-based organizations in Shenzhen (51.6% at Month 6 vs. 11.4% at baseline, *p* < 0.001), and HIV testing at public hospitals/CDC in Shenzhen (35.2% at Month 6 vs. 20.6% at baseline, *p* < 0.001) (Table 3).

### Changes in Sexual Behavior at Baseline and Follow-Up Surveys

As compared to baseline, more participants engaged in condomless anal intercourse with regular male sex partners (34.5% at Month 6 vs. 24.0% at baseline, *p* = 0.002) and male sex workers (12.9% at Month 6 vs. 2.4% at baseline, *p* < 0.001), and sexualized drug use (21.6% at Month 6 vs. 12.1% at baseline, *p* = 0.001) at Month 6. Within individuals, 19.4, 11.0, and 4.3% increased condomless anal intercourse with regular male sex partners, non-regular male sex partners, and male sex workers over the study period, and 14.0% increased sexualized drug use (Tables 2, 3).

**Table 2:** Barriers to take up HIV testing caused by COVID-19 and its control measures and changes in sexual behaviors (*n* = 412).

	**%^a^**
**Predisposing factors**	
**Condomless anal intercourse with regular male sex partners**	
With such behavior at baseline only	9.0
No such behavior either at baseline or follow-up	56.6
With such behavior both at baseline and follow-up	15.0
With such behavior at follow-up only	19.4
**Condomless anal intercourse with non-regular male sex partners**	
With such behavior at baseline only	7.0
No such behavior either at baseline or follow-up	76.7
With such behavior both at baseline and follow-up	5.3
With such behavior at follow-up only	11.0
**Condomless anal intercourse with male sex workers**	
With such behavior at baseline only	2.0
No such behavior either at baseline or follow-up	93.2
With such behavior both at baseline and follow-up	0.5
With such behavior at follow-up only	4.3
**Sexualized drug use**	
With such behavior at baseline only	4.6
No such behavior either at baseline or follow-up	73.8
With such behavior both at baseline and follow-up	7.5
With such behavior at follow-up only	14.0
**Enabling factors**	
**Barriers to take up HIV testing caused by COVID-19 and its control measures during the follow-up period, % Yes**	
HIV testing service providers suspended their services	12.4
HIV testing service providers reduced their service hours	14.3
Difficult to obtain HIV self-testing kits	10.4
History of home/centralized quarantine	12.6
You avoided unnecessary travel and tried to stay at home	54.6
You avoided going to crowded places	60.9
**Reinforcing factors**	
Your male sex partners or friends suggested you not to take up HIV testing	16.5

a *Multiple imputation was performed to replace missing values at Month 6. Markov chain Monte Carlo methods were used for data with an arbitrary pattern of missing values, while Monotone methods were used for data having a monotone pattern of missing values. Predictors included baseline background characteristics and baseline value of the variable with missing values at Month 6*.

**Table 3:** Comparing HIV testing behaviors and sexual behaviors in the past 6 months measured at baseline and follow-up (*n* = 412).

	**Baseline**	**Follow-up ^a^**	***P*** **-values**
	**% Yes**	**% Yes**	
**Sexual behaviors**			
Condomless anal intercourse with regular male sex partners	24.0	34.5	**0.002**
Condomless anal intercourse with non-regular male sex partners	12.4	16.2	0.34
Condomless anal intercourse with male sex workers	2.4	12.9	**<0.001**
Sexualized drug use	12.1	21.6	**0.001**
**Utilization of HIV prevention services**			
HIV testing at community-based organization in Shenzhen	11.4	51.6	**<0.001**
HIV testing at public hospitals or CDC in Shenzhen	20.6	35.2	**<0.001**
HIV testing at private hospitals in Shenzhen	4.9	13.1	**<0.001**
HIV testing at other organizations in Shenzhen	9.2	17.2	**0.01**
HIV testing outside Shenzhen	18.0	23.3	0.16
Home-based HIV self-testing	42.7	52.4	**0.001**
Any type of HIV testing	59.2	77.9	**<0.001**

a *Multiple imputation was performed to replace missing values at Month 6. Markov chain Monte Carlo methods were used for data with an arbitrary pattern of missing values, while Monotone methods were used for data having a monotone pattern of missing values. Predictors included baseline background characteristics and baseline value of the variable with missing values at Month 6. The bold values indicate the values of p < 0.05 which are statistically significant*.

### Factors Associated With Uptake of any Type of HIV Testing During the Follow-Up Period

In univariate analysis, current employment status and monthly personal income were associated with uptake of any type of HIV testing during the follow-up period (Table 4).

**Table 4:** Associations between baseline sociodemographic and uptake of any type of HIV testing during the follow-up period (*n* = 412).

	**OR (95%CI)**	***P*** **-values**
**Sociodemographic**		
**Age group (years)**		
18–24	1.0	
25–30	0.87 (0.51, 1.50)	0.62
31–40	1.35 (0.63, 2.89)	0.44
>40	3.07 (0.67, 13.94)	0.15
**Current relationship status**		
Currently single	1.0	
Married or cohabited with a man	0.64 (0.35, 1.17)	0.15
Married or cohabited with a woman	1.73 (0.38, 7.87)	0.48
**Highest education level attained**		
Junior high or below	1.0	
Senior high or equivalent	1.13 (0.41, 3.10)	0.82
College or above	0.42 (0.18, 1.00)	0.05
Others	0.56 (0.15, 2.83)	0.56
**Current employment status**		
Full-time	1.0	
Part-time/unemployed /retired/students/others	0.55 (0.33, 0.91)	**0.02**
**Monthly personal income, Chinese Yuan (US dollar)**		
No fixed income	1.0	
Below 3,000 (461.4)	1.98 (0.71, 5.47)	0.19
3,000–4,999 (461.4–768.9)	2.79 (1.02, 7.67)	**0.04**
5,000–6,999 (769.1–1076.5)	1.16 (0.44, 3.06)	0.77
7,000–9,999 (1076.7–1537.9)	1.25 (0.49, 3.16)	0.64
10,000 or above (1538.1)	0.88 (0.28, 2.74)	0.82
Refuse to disclose	3.68 (0.69, 19.63)	0.13
**Sexual orientation**		
Homosexual	1.0	
Bisexual	2.12 (0.95, 4.74)	0.07
Heterosexual	2.05 (0.45, 9.36)	0.36
Uncertain	2.55 (0.57, 11.36)	0.22
**Source of recruitment**		
Outreaching in venues	1.0	
Online recruitment	0.79 (0.46, 1.36)	0.40
Peer referral	1.31 (0.79, 2.17)	0.30

*Multiple imputation was performed to replace missing values at Month 6. Markov chain Monte Carlo methods were used for data with an arbitrary pattern of missing values, while Monotone methods were used for data having a monotone pattern of missing values. Predictors included baseline background characteristics and baseline value of the variable with missing values at Month 6. OR: crude odds ratios obtained from two-level logistic regression models (level 1: sources of recruitment, level 2: individual participants). CI, confidence interval. The bold values indicate the values of p < 0.05 which are statistically significant*.

After adjusting for age group, education level, current employment status, and monthly personal income, two predisposing factors were associated with higher uptake of HIV testing during the follow-up period. They were: (1) condomless anal intercourse with male non-regular male sex partners at follow-up only (AOR: 5.29, 95%CI: 1.27, 22.01) and (2) sanitizing before and after sex at baseline (AOR: 1.26, 95%CI: 1.02, 1.47). Regarding enabling factors, utilization of HIV testing (AOR: 3.90, 95%CI: 2.27, 6.69) and STI testing (AOR: 2.43, 95%CI: 1.20, 4.93) 6 months prior to the baseline survey was associated with higher uptake of HIV testing during the follow-up period. Having the experience that HIV testing service providers reduced service hours during the follow-up period was also positively associated with the dependent variable (AOR: 3.45, 95%CI: 1.26, 9.41) (Table 5).

**Table 5:** Factors associated with uptake of any type of HIV testing during the follow-up period (among participants who completed both surveys, *n* = 297).

	**OR (95%CI)**	***P*** **-values**	**AOR (95%CI)**	***P*** **-values**
**Predisposing factors**				
**Condomless anal intercourse with regular male sex partners**				
No such experience either at baseline or follow-up/With such experience at baseline only	1.0		1.0	
With such experience both at baseline and follow-up	1.36 (0.62, 2.99)	0.44	1.43 (0.63, 3.24)	0.38
With such experience at follow-up only	1.77 (0.81, 3.84)	0.15	1.73 (0.71, 4.27)	0.22
**Condomless anal intercourse with non-regular male sex partners**				
No such experience either at baseline or follow-up/With such experience at baseline only	1.0		1.0	
With such experience both at baseline and follow-up	3.05 (0.62, 14.90)	0.17	2.72 (0.56, 13.09)	0.21
With such experience at follow-up only	3.96 (1.18, 13.38)	**0.03**	5.29 (1.27, 22.01)	**0.02**
**Condomless anal intercourse with male sex workers**				
No such experience either at baseline or follow-up/With such experience at baseline only	1.0		1.0	
With such experience both at baseline and follow-up	0.29 (0.02, 4.68)	0.38	0.16 (0.01, 2.89)	0.22
With such experience at follow-up only	2.32 (0.52, 10.28)	0.27	2.15 (0.45, 10.35)	0.34
**Sexualized drug use**				
No such experience either at baseline or follow-up/With such experience at baseline only	1.0		1.0	
With such experience both at baseline and follow-up	2.13 (0.64, 7.05)	0.22	1.83 (0.55, 6.08)	0.32
With such experience at follow-up only	2.02 (0.85, 4.80)	0.11	2.10 (0.77, 5.74)	0.15
**COVID-19 preventive measures during sexual behaviors (February to July 2020)**				
Only having sex with my partner who has had sex with me before	0.92 (0.78, 1.08)	0.30	0.96 (0.80, 1.14)	0.63
Avoiding group sex	0.87 (0.76. 1.01)	0.06	0.92 (0.79, 1.06)	0.25
Only having sex at home	0.91 (0.77, 1.08)	0.28	0.94 (0.78, 1.14)	0.53
Asking your partner if they have COVID-19 symptoms	1.05 (0.89, 1.24)	0.59	1.10 (0.92, 1.31)	0.31
Avoiding kissing during sex	1.14 (0.95, 1.35)	0.16	1.12 (0.93, 1.35)	0.22
Washing hands before and after sex	1.08 (0.92, 1.26)	0.33	1.08 (0.91, 1.28)	0.41
Sanitizing before and after sex	1.18 (1.02, 1.38)	**0.03**	1.26 (1.02, 1.47)	**0.01**
**Psychosocial variables related to COVID-19 and HIV testing**				
Worry about being infected with COVID-19 when undertaking HIV testing	1.08 (0.90, 1.30)	0.42	1.05 (0.87, 1.28)	0.61
Perceived effectiveness of COVID-19 control measures taken by organizations providing HIV testing	1.13 (0.91, 1.41)	0.28	1.10 (0.86, 1.39)	0.44
Level of panic of COVID-19 infections	0.99 (0.78, 1.27)	0.95	0.95 (0.72, 1.25)	0.71
**Enabling factors**				
**HIV testing in the past 6months (February to July 2020)**				
No	1.0		1.0	
Yes	3.17 (1.93, 5.20)	**<0.001**	3.90 (2.27, 6.69)	**<0.001**
**Testing for STI in the past 6 months (February to July 2020)**				
No	1.0		1.0	
Yes	2.13 (1.11, 4.11)	**0.02**	2.43 (1.20, 4.93)	**0.01**
**Utilization of other HIV/STI prevention services in the past 6 months (February to July 2020)**				
No	1.0		1.0	
Yes	1.66 (0.98, 2.82)	0.06	1.68 (0.95, 2.96)	0.07
**Use of pre-exposure prophylaxis in the past 6 months (February to July 2020)**				
No	1.0		1.0	
Yes	1.80 (0.50, 6.54)	0.37	2.03 (0.51, 8.08)	0.31
**Barriers to take up HIV testing caused by COVID-19 and its control measures during the follow-up period**				
HIV testing service providers suspended their services	2.88 (0.95, 8.73)	0.06	2.79 (0.81, 9.60)	0.10
HIV testing service providers reduced their service hours	1.95 (1.12, 7.77)	**0.03**	3.45 (1.26, 9.41)	**0.02**
Difficult to obtain HIV self-testing kits	1.64 (0.55, 4.86)	0.37	1.63 (0.55, 4.85)	0.37
History of home/centralized quarantine	1.59 (0.37, 6.90)	0.50	1.60 (0.33, 7.68)	0.52
You avoided unnecessary travel and tried to stay at home	0.82 (0.49, 1.39)	0.47	0.85 (0.47, 1.51)	0.57
You avoided going to crowdeded places	1.33 (0.81, 2.18)	0.26	1.36 (0.78, 2.37)	0.28
**Reinforcing factors**				
Your male sex partners or friends suggested you not to take up HIV testing	0.92 (0.22, 3.84)	0.91	1.19 (0.39, 3.68)	0.75

*Multiple imputation was performed to replace missing values at Month 6. Markov chain Monte Carlo methods were used for data with an arbitrary pattern of missing values, while Monotone methods were used for data having a monotone pattern of missing values. Predictors included baseline background characteristics and baseline value of the variable with missing values at Month 6.vOR, crude odds ratios obtained from two-level logistic regression models (level 1: sources of recruitment, level 2: individual participants); AOR, adjusted odds ratios, odds ratios obtained from two-level logistic regression models (level 1: sources of recruitment, level 2: individual participants) after adjusting for age group, education level, current employment status, and monthly income level; CI, confidence interval. The bold values indicate the values of p < 0.05 which are statistically significant*.

## Discussion

To our knowledge, this is one of the first prospective cohort studies to investigate the changes in HIV testing utilization among MSM before and after the COVID-19 pandemic received initial control. As compared to baseline data, significant increases in the uptake of any type of HIV testing, facility-based HIV testing (e.g., those performed at community-based organizations, public and private hospitals), and HIV self-testing were observed at follow-up. COVID-19 preventive measures during sexual behaviors and HIV-related service utilization at baseline and changes in sexual behaviors and barriers caused by COVID-19 and its control measures over the study period were associated with HIV testing uptake during the follow-up period. The use of the PRECEDE model provided a more comprehensive overview of factors influencing HIV testing uptake and expanded the application of such a model.

About 60% of the sampled MSM used any type of HIV testing between February and July 2020. Such uptake rate was slightly lower than the level before the COVID-19 outbreak (about 70%) (35, 36). Our findings were in line with previous studies investigating the potential impact of COVID-19 on HIV testing service utilization in China (12, 13). However, the magnitude of such impact might be smaller than that of other countries (8–11). In line with our hypothesis, HIV testing uptake had rebounded after the COVID-19 received control. A significant increase in the uptake of any type of HIV testing was observed during the study period. At follow-up, 77.9% of participants received any type of HIV testing in the past 6 months. Such uptake rate was similar to that observed in the time before the COVID-19 outbreak in China (35, 36).

The changes in the utilization of specific types of HIV testing provided a more comprehensive picture of the impacts of the COVID-19 pandemic. Before the COVID-19 outbreak, community-based organizations, public hospitals, and CDC provided over 80% of HIV testing services for MSM, while private hospitals and other organizations provided almost 30% of HIV testing services for them (37). Our findings suggested that HIV testing services provided by these organizations were most affected by the COVID-19 pandemic and its control measures in China, as only 11.4, 20.6, 4.9, and 9.2% of our participants used facility-based HIV testing at community-based organizations, public hospitals/CDC, private hospitals and other organizations between February and July 2020, respectively. Before the COVID-19 pandemic received initial control, MSM in China mainly relied on home-based HIV self-testing. The utilization of HIV self-testing during February and July 2020 (42.7%) was higher than that observed in the pre-pandemic period (20.3–39.7%) (38–41). Many users of the facility-based HIV testing might shift to HIV self-testing during the COVID-19 pandemic (13). Promotion and implementation of HIV self-testing among MSM have been quite successful in China, which might mitigate the negative impact of the COVID-19 pandemic on HIV testing service utilization (42). After the COVID-19 pandemic received initial control, the largest increase was observed for HIV testing at community-based organizations, followed by HIV testing at public hospitals/CDC, private hospitals, and other organizations. It was encouraging that the use of HIV self-testing also increased over time. It is possible that many MSM tried HIV self-testing for the first time during the pandemic and found it an appealing alternative option to facility-based HIV testing (13).

Our findings suggested some reasons to explain the changes in HIV testing utilization and provided some empirical implications to enhance HIV testing coverage among MSM in the “post-pandemic era.” Similar to the findings of a previous study, MSM without a full-time job were less likely to use HIV testing during the follow-up period (43). In addition, lower income was also a barrier to use HIV testing during the follow-up period. Future programs should pay more attention to MSM with lower socio-economic status.

Changes in condomless anal intercourse with non-regular male sex partners was a significant predisposing factor of HIV testing utilization during the follow-up period. As compared to those without/reduced condomless anal intercourse with non-regular male sex partners during the follow-up period, an increase in condomless anal intercourse with non-regular male sex partners was associated with higher HIV testing uptake. It is possible that those with an increase in condomless anal intercourse with non-regular male sex partners would perceive a higher risk of HIV transmission, which motivated them to take up HIV testing. Although condomless anal intercourse with regular male sex partners, condomless anal intercourse with male sex workers, and sexualized drug use significantly increased over the follow-up period, such increase did not motivate them to receive HIV testing. Health communication messages that condomless anal intercourse with regular male sex partners and sexualized drug use are independent risk factors of HIV acquisition should be disseminated to MSM to increase their awareness (44, 45). Use of COVID-19 preventive measures during sexual behaviors, such as sanitizing before and after sex, was associated with higher uptake of HIV testing during the follow-up period. It is possible that MSM who adopted this preventive measure had higher motivation and self-efficacy to protect themselves (46, 47).

Utilization of HIV, STI, and other HIV/STI prevention services prior to the baseline survey was associated with higher uptake of HIV testing during the follow-up period. Previous studies suggested that frequent users of HIV testing were more likely to take up HIV testing again (48, 49). MSM who had utilized STI testing and other HIV/STI prevention services might be more familiar with the service providers and hence had a higher level of trust (50). The trust of service providers was a facilitator of receiving HIV testing (51). In contrast to our hypothesis, the exposure to HIV testing services with reduced service hours was positively associated with the outcome. One possible explanation is that those who were proactively seeking HIV testing would know about the changes in HIV testing service hours, while those who did not seek might be less likely to know about it.

Our study has some policy and program implications. After the control of the COVID-19 pandemic, MSM would increase sexual activities and hence have a stronger need for HIV testing. Therefore, local government and service providers should resume their workforce and working hours for HIV prevention services. Our findings also suggested that facility-based HIV testing services were most affected during the COVID-19 outbreak. Local service providers should consider promoting home-based HIV self-testing and relevant supporting services (e.g., online counseling support and referral services for HIV self-testing users) to mitigate the negative impact caused by the COVID-19 pandemic.

This study had a number of limitations. First, participants were recruited by non-probability sampling in the absence of a sampling frame. The findings may not be representative of MSM in Shenzhen. Since we only conducted the study in one Chinese city, caution should be taken when generalizing the findings to other parts of China. As compared to other less developed cities in China, more organizations are providing HIV testing services for MSM in Shenzhen. Moreover, given the higher income level, MSM in Shenzhen would have lower financial barriers to use chargeable HIV testing services provided by private clinics or to purchase HIV self-testing kits. Therefore, the findings in this article could not apply to other Chinese cities with a general or lower economy. The COVID-19 pandemic might have a greater impact on HIV testing services in other smaller or less developed Chinese cities. Second, the results were self-reported and subject to social desirability bias. Participants might over-report HIV uptake and under-report sexual risk behaviors. Third, attribution bias existed. As compared to those who were followed up, dropouts had lower utilization of HIV testing and were less likely to use COVID-19 preventive behaviors during sexual intercourse at baseline. Therefore, the prevalence of HIV testing uptake at follow-up might be overestimated. Fourth, we were not able to obtain the characteristics of participants who refused to join the study. Participants and refusals might have different characteristics. Selection bias existed. However, the response rate was relatively high as compared to other published studies in China. Finally, we did not find validated measurements based on the PRECEDE model that were applicable to HIV testing behaviors under the COVID-19 pandemic. Therefore, we self-constructed the measurements based on panel discussion involving public health researcher, epidemiologist, psychologist, local MSM, and staff of local community-based organizations.

## Conclusions

As compared to the baseline data, the uptake of any type of HIV testing, facility-based HIV testing at community-based organizations, public hospitals/CDC, private hospitals, other organizations, and HIV self-testing increased significantly at follow-up. HIV testing utilization among MSM might rebound to the level before the COVID-19 outbreak after the pandemic received initial control in China. Using the PRECEDE model, this study offered a comprehensive overview to identify potential reasons that can influence the uptake of HIV testing among Chinese MSM in the post-pandemic era.

## Data Availability Statement

The data presented in this study are available from the corresponding author upon request. The data are not publicly available as they contain sensitive personal behaviors.

## Ethics Statement

The studies involving human participants were reviewed and approved by the Institutional Review Boards of Longhua District Center for Disease Control and Prevention (CDC) (reference: 2021009). Informed consent was obtained from all participants involved in the study.

## Author Contributions

KZ and ZW: conceptualization, methodology, and supervision. KZ, HCa, HCh, TH, YC, and XZ: data curation and project administration. ZW, SC, and PC: formal analysis. ZW, SC, YF, and PC: writing-original draft preparation and writing-review and editing. All authors have read and agreed to the published version of the manuscript.

## Funding

This study was funded by the High-Level Project of Medicine in Longhua, Shenzhen (HLPM201907020105) and the Key Discipline of Infectious Diseases Control and Prevention of Longhua (grant number 2020-2014).

## Conflict of Interest

The authors declare that the research was conducted in the absence of any commercial or financial relationships that could be construed as a potential conflict of interest.

## Publisher's Note

All claims expressed in this article are solely those of the authors and do not necessarily represent those of their affiliated organizations, or those of the publisher, the editors and the reviewers. Any product that may be evaluated in this article, or claim that may be made by its manufacturer, is not guaranteed or endorsed by the publisher.

## References

[1] Dong MJ Peng B Liu ZF Ye QN Liu H Lu XL. The prevalence of HIV among MSM in China: a large-scale systematic analysis. BMC Infect Dis. (2019) 19:1000. 10.1186/s12879-019-4559-1 31775654 PMC6880607

[2] Zhang W Xu JJ Zou H Zhang J Wang N Shang H. incidence and associated risk factors in men who have sex with men in Mainland China: an updated systematic review and meta-analysis. Sex Health. (2016) 13:373–82. 10.1071/SH16001 27294689

[3] Tsui H Lau JT Xiang W Gu J Wang Z. Should associations between HIV-related risk perceptions and behaviors or intentions be positive or negative? PLoS ONE. (2012) 7:e52124. 10.1371/journal.pone.0052124 23284896 PMC3526527

[4] Gu J Bai Y Lau JT Hao Y Cheng Y Zhou R. Social environmental factors and condom use among female injection drug users who are sex workers in China. AIDS Behav. (2014) 18:S181–91. 10.1007/s10461-013-0434-z 23443978 PMC3749245

[5] Lau JT Gu J Tsui HY Chen H Holroyd E Wang R. Prevalence and associated factors of condom use during commercial sex by female sex workers who were or were not injecting drug users in China. Sex Health. (2012) 9:368–76. 10.1071/SH11108 22877597

[6] Zhou J Chen J Goldsamt L Wang H Zhang C Li X. Testing and associated factors among men who have sex with men in Changsha, China. J Assoc Nurses AIDS Care. (2018) 29:932–41. 10.1016/j.jana.2018.05.003 29861317 PMC6204106

[7] World Health Organization. Coronavirus Disease (COVID-2019) Situation Reports. Available online at: https://www.who.int/emergencies/diseases/novel-coronavirus-2019/situation-reports (accessed November 17, 2020).

[8] Ejima K Koizumi Y Yamamoto N Rosenberg M Ludema C Bento AI. HIV testing by public health centers and municipalities and new HIV cases during the COVID-19 pandemic in Japan. J Acquir Immune Defic Syndr. (2021) 87:e182. 10.1101/2020.10.16.20213959 33625066 PMC8126475

[9] Chow EPF Ong JJ Denham I Fairley CK HIV. Testing and diagnoses during the COVID-19 pandemic in Melbourne, Australia. J Acquir Immune Defic Syndr. (2021) 86:e114–5. 10.1097/QAI.0000000000002604 33346567 PMC7901531

[10] Santos GM Ackerman B Rao A Wallach S Ayala G Lamontage E. Economic, mental health, HIV prevention and HIV treatment impacts of COVID-19 and the COVID-19 response on a global sample of cisgender gay men and other men who have sex with men. AIDS Behav. (2021) 25:311–21. 10.1007/s10461-020-02969-0 32654021 PMC7352092

[11] Sanchez TH Zlotorzynska M Rai M Baral SD. Characterizing the impact of COVID-19 on men who have sex with men across the United States in April, 2020. AIDS Behav. (2020) 24:2024–32. 10.1007/s10461-020-02894-2 32350773 PMC7189633

[12] Booton RD Fu G MacGregor L Li J Ong JJ Tucker JD. The impact of disruptions due to COVID-19 on HIV transmission and control among men who have sex with men in China. J Int AIDS Soc. (2021) 24:e25697. 10.1002/jia2.25697 33821553 PMC8022092

[13] Jiang H Xie Y Xiong Y Zhou Y Lin K Yan Y. HIV self-testing partially filled the HIV testing gap among men who have sex with men in China during the COVID-19 pandemic: results from an online survey. J Int AIDS Soc. (2021) 24:e25737. 10.1002/jia2.25737 34036750 PMC8150052

[14] Green LW Kreuter MW. Health Promotion Planning: An Educational and Ecological Approach. 3rd ed. Mountain View, CA: Mayfield (1999).

[15] Ponticiello M Mwanga-Amumpaire J Tushemereirwe P Nuwagaba G King R Sundararajan R. “Everything is a mess”: how COVID-19 is impacting engagement with HIV testing services in rural southwestern Uganda. AIDS Behav. (2020) 24:3006–9. 10.1007/s10461-020-02935-w 32451939 PMC7246959

[16] Lagat H Sharma M Kariithi E Otieno G Katz D Masyuko S. Impact of the COVID-19 pandemic on HIV testing and assisted partner notification services, western kenya. AIDS Behav. (2020) 24:3010–3. 10.1007/s10461-020-02938-7 32488552 PMC7265868

[17] Mhango M Chitungo I Dzinamarira T. COVID-19 lockdowns: impact on facility-based HIV testing and the case for the scaling up of home-based testing services in Sub-Saharan Africa. AIDS Behav. (2020) 24:3014–6. 10.1007/s10461-020-02939-6 32488551 PMC7265663

[18] Suen YT Chan RCH Wong EMY. An exploratory study of factors associated with difficulties in accessing HIV services during COVID-19 among Chinese gay and bisexual men in Hong Kong. Int J Infect Dis. (2021) 106:358-62. 10.1016/j.ijid.2021.04.005 33845197 PMC8047335

[19] Wang Z Mo PKH Ip M Fang Y Lau JTF. Uptake and willingness to use PrEP among Chinese gay, bisexual and other men who have sex with men with experience of sexualized drug use in the past year. BMC Infect Dis. (2020) 20:299. 10.1186/s12879-020-05024-4 32321442 PMC7178573

[20] Kurtz SP. Post-circuit blues: motivations and consequences of crystal meth use among gay men in Miami. AIDS Behav. (2005) 9:63–72. 10.1007/s10461-005-1682-3 15812614

[21] Wang Z Yang X Mo PKH Fang Y Ip TKM Lau JTF. Influence of social media on sexualized drug use and Chemsex among Chinese men who have sex with men: observational prospective cohort study. J Med Internet Res. (2020) 22:e17894. 10.2196/17894 32706705 PMC7414399

[22] Zhao P Liu L Zhang Y Cheng H Cao B Liu C. The interaction between HIV testing social norms and self-efficacy on HIV testing among Chinese men who have sex with men: results from an online cross-sectional study. BMC Infect Dis. (2018) 18:541. 10.1186/s12879-018-3454-5 30376818 PMC6208016

[23] Yuan J Cao B Zhang C Chan PS-f Xin M Fang Y. Changes in compliance with personal preventive measures and mental health status among Chinese factory workers during the COVID-19 pandemic: an observational prospective cohort study. Front Public Health. (2022) 10:831456. 10.3389/fpubh.2022.831456 35359764 PMC8960195

[24] Wang Z Lau JTF She R Ip M Jiang H Ho SPY. Behavioral intention to take up different types of HIV testing among men who have sex with men who were never-testers in Hong Kong. AIDS Care. (2018) 30:95–102. 10.1080/09540121.2017.1338659 28637357

[25] Wang Z Lau JTF Ip M Ho SPY Mo PKH Latkin C. Randomized controlled trial evaluating efficacy of promoting a home-based HIV self-testing with online counseling on increasing HIV testing among men who have sex with men. AIDS Behav. (2018) 22:190–201. 10.1007/s10461-017-1887-2 28831616

[26] Wang Z Fang Y Yaemim N Jonas KJ Chidgey A Ip M. Factors predicting uptake of sexually transmitted infections testing among men who have sex with men who are “pre-exposure prophylaxis tourists”-an observational prospective cohort study. Int J Environ Res Public Health. (2021) 18:3582. 10.3390/ijerph18073582 33808349 PMC8036909

[27] He J Xu HF Cheng WB Zhang SJ Gu J Hao YT. Intimate relationship characteristics as determinants of HIV risk among men who have sex with regular male sex partners: a cross-sectional study in Guangzhou, China. BMC Infect Dis. (2018) 18:150. 10.1186/s12879-018-3044-6 29606100 PMC5879993

[28] Wang X Wang Z Jiang X Li R Wang Y Xu G. cross-sectional study of the relationship between sexual compulsivity and unprotected anal intercourse among men who have sex with men in shanghai, China. BMC Infect Dis. (2018) 18:465. 10.1186/s12879-018-3360-x 30219033 PMC6139151

[29] Zhang K Chan PS Chen S Fang Y Cao H Chen H. Factors predicting COVID-19 vaccination uptake among men who have sex with men in China: an observational prospective cohort study. Front Med. (2022) 9:838973. 10.3389/fmed.2022.838973 35360721 PMC8963419

[30] Ye R Liu C Tan S Li J Simoni JM Turner D. Factors associated with past HIV testing among men who have sex with men attending university in China: a cross-sectional study. Sex Health. (2021) 18:58–63. 10.1071/SH20088 33639685 PMC10767712

[31] Lau JT Gu J Tsui HY Wang Z. Prevalence and associated factors of intention to participate in HIV voluntary counseling and testing for the first time among men who have sex with men in Hong Kong, China. Prev Med. (2013) 57:813–8. 10.1016/j.ypmed.2013.09.005 24045009

[32] Han J Jia P Huang Y Gao B Yu B Yang S. Association between social capital and mental health among older people living with HIV: the Sichuan older HIV-infected cohort study (SOHICS). BMC Public Health. (2020) 20:581. 10.1186/s12889-020-08705-6 32345273 PMC7189431

[33] Pan Y Fang Y Xin M Dong W Zhou L Hou Q. Self-reported compliance with personal preventive measures among Chinese factory workers at the beginning of work resumption following the COVID-19 outbreak: cross-sectional survey study. J Med Internet Res. (2020) 22:e22457. 10.2196/22457 32924947 PMC7527164

[34] Zhang KC Fang Y Cao H Chen H Hu T Chen Y. Behavioral intention to receive a COVID-19 vaccination among Chinese factory workers: cross-sectional online survey. J Med Internet Res. (2021) 23:e24673. 10.2196/24673 33646966 PMC7945977

[35] Wei L Chen L Zhang H Yang Z Liu S Tan W. Relationship between gay app use and HIV testing among men who have sex with men in Shenzhen, China: a serial cross-sectional study. BMJ Open. (2019) 9:e028933. 10.1136/bmjopen-2019-028933 31446409 PMC6721534

[36] Liu Y Qian HZ Ruan Y Wu P Osborn CY Jia Y. Frequent HIV testing: impact on HIV risk among Chinese men who have sex with men. J Acquir Immune Defic Syndr. (2016) 72:452–61. 10.1097/QAI.0000000000001001 27003496 PMC4925192

[37] Han L Wei C Muessig KE Bien CH Meng G Emch ME. HIV test uptake among MSM in China: implications for enhanced HIV test promotion campaigns among key populations. Glob Public Health. (2017) 12:31–44. 10.1080/17441692.2015.1134612 26785328 PMC4955642

[38] Han L Bien CH Wei C Muessig KE Yang M Liu F. HIV self-testing among online MSM in China: implications for expanding HIV testing among key populations. J Acquir Immune Defic Syndr. (2014) 67:216–21. 10.1097/QAI.0000000000000278 24991972 PMC4162828

[39] Ren XL Wu ZY Mi GD McGoogan J Rou KM Zhao Y. Uptake of HIV Self-testing among men who have sex with men in Beijing, China: a cross-sectional study. Biomed Environ Sci. (2017) 30:407–17. 10.1186/s40249-017-0326-y 28705264

[40] Hong H Shi HB Jiang HB Dong HJ Shen YL. Prevalence and associated factors of HIV self-testing among men who have sex with men in Ningbo, China: a cross-sectional study. AIDS Res Ther. (2021) 18:14. 10.1186/s12981-021-00339-x 33879191 PMC8056656

[41] Qin Y Liu F Tang W Tang S Liu C Mao J. HIV self-testing among high-risk men who have sex with men in China: a cross-sectional study. Lancet. (2016) 388:S76. 10.1016/S0140-6736(16)32003-7

[42] Tang W Wu D. Opportunities and challenges for HIV self-testing in China. Lancet HIV. (2018) 5:e611–e2. 10.1016/S2352-3018(18)30244-3 30213725 PMC7250046

[43] Maulsby CH Ratnayake A Hesson D Mugavero MJ Latkin CA. A scoping review of employment and HIV. AIDS Behav. (2020) 24:2942–55. 10.1007/s10461-020-02845-x 32246357 PMC7716244

[44] Hao C Yan H Yang H Huan X Guan W Xu X. The incidence of syphilis, HIV and HCV and associated factors in a cohort of men who have sex with men in Nanjing, China. Sex Transm Infect. (2011) 87:199–201. 10.1136/sti.2010.042903 21262785

[45] Maxwell S Shahmanesh M Gafos M. Chemsex behaviours among men who have sex with men: A systematic review of the literature. Int J Drug Policy. (2019) 63:74–89. 10.1016/j.drugpo.2018.11.014 30513473

[46] Shilo G Mor Z. COVID-19 and the changes in the sexual behavior of men who have sex with men: results of an online survey. J Sex Med. (2020) 17:1827–34. 10.1016/j.jsxm.2020.07.085 32883631 PMC7416743

[47] Calabrò RS. Sexual behavior during the Covid-19 pandemic: it's telecounseling time! *Innov Clin Neurosci*. (2021) 18:8–9. 34150355 PMC8195564

[48] Chan PS Chidgey A Lau J Ip M Lau JTF Wang Z. Effectiveness of a novel HIV self-testing service with online real-time counseling support (HIVST-Online) in increasing HIV testing rate and repeated HIV testing among men who have sex with men in Hong Kong: results of a pilot implementation project. Int J Environ Res Public Health. (2021) 18:15. 10.3390/ijerph18020729 33467770 PMC7830557

[49] Zhao Y Bromberg DJ Khoshnood K Sheng Y. Factors associated with regular HIV testing behavior of MSM in China: a cross-sectional survey informed by theory of triadic influence. Int J STD AIDS. (2020) 31:1340–51. 10.1177/0956462420953012 33081648

[50] Eaton LA Driffin DD Kegler C Smith H Conway-Washington C White D. The role of stigma and medical mistrust in the routine health care engagement of black men who have sex with men. Am J Public Health. (2015) 105:e75–82. 10.2105/AJPH.2014.302322 25521875 PMC4318301

[51] Gu J Lau JT Wang Z Wu AM Tan X. Perceived empathy of service providers mediates the association between perceived discrimination and behavioral intention to take up HIV antibody testing again among men who have sex with men. PLoS ONE. (2015) 10:e0117376. 10.1371/journal.pone.0117376 25693179 PMC4333296

